# Immunotherapy for acute leukemia

**DOI:** 10.18632/aging.100768

**Published:** 2015-06-24

**Authors:** Michael Boyiadzis, Theresa L Whiteside, Steven Z Pavletic

**Affiliations:** University of Pittsburgh Cancer Institute, University of Pittsburgh School of Medicine, Pittsburgh, PA, USA

Allogeneic hematopoetic cell transplantation (allo-HCT) can be curative for patients with acute leukemia [1]. The anti-leukemia activity relies on the graft-*versus*-leukemia (G*V*L) effects that are elicited after allo-HCT, suggesting that anti-tumor immunity may be responsible for eradicating leukemia and preventing subsequent relapse. However, allo-HCT is associated with significant morbidity and mortality resulting from toxicity of dose-intensive conditioning regimens and graft-*versus*-host disease (G*V*HD), limiting allo-HCT to younger and medically fit patients. Over the last two decades, non-myeloablative conditioning regimens have been developed to reduce toxicity and allow the use of allo-HCT in older patients [2]. These regimens are designed to provide sufficient immune suppression just to allow donor cell engraftment and induction of G*V*L effects as the primary therapy. Despite the more frequent use of non-myeloablative allo-HCT in older patients, G*V*HD still remains a major challenge.

Several retrospective studies have demonstrated that both acute and chronic G*V*HD are associated with a decreased incidence of relapses [3, 4]. However, effects of chronic G*V*HD on relapse are difficult to separate from acute G*V*HD effects, because most cases of chronic G*V*HD occur within the first year after transplant, at the time when acute G*V*HD is still active. Also, since majority leukemia relapses occur within the first year after allo-HCT, there is possibility of little net-additive chronic G*V*HD anti-leukemic benefit when it comes to affecting late (> 12 months) relapses. In a recent study, we evaluated the effects of chronic G*V*HD on late relapse in 7,489 allo-HCT recipients who were alive one year after HCT [5]. The protective effect of chronic GVHD on late relapse was present only in patients with chronic myeloid leukemia (CML) and was not seen in patients with acute leukemia. Although providing protection from late relapse in patients with CML, chronic G*V*HD was associated with higher risk of treatment-related mortality and inferior overall survival for all diseases. These results suggest that development of more assertive strategies for chronic G*V*HD therapy and prevention should be justified to decrease treatment related mortality after allo-HCT.

Within the first year after allo-HCT both treatment related mortality and leukemic relapse can be as high as 30–40% in high risk patients [6]. In view of these results, the question arise is whether we can mimic the G*V*L effects of donor cells by enhancing the antitumor effects of the patients' immune system and avoiding allo-HCT and its associated complications. The answer to this question is “not yet.” The prospect is that immunotherapy administered in lieu of allo-HCT will induce anti-leukemic immunity strong enough to eliminate leukemia and also make such treatments more tolerable in older patients. Clinical trials studying different immune therapies in patients with acute leukemia are on-going and are expected to provide the much needed answers about most effective approaches to generating and sustaining anti-leukemia immunity. The current armamentarium of immune approaches in acute leukemia includes antibodies, cytokines, cell therapies, vaccines and check point inhibitors, among others [7]. More importantly, combinations of different immune therapeutic approaches are being investigated to maximize the host anti-leukemia effects. Multiples antigens, including CD33, CD123 and CCL-1, represent potential targets for antibody-based therapy in acute leukemia. To further enhance the efficacy of antibody-based therapies, bispecific T-cell engager (BITE) antibodies (Abs) have been introduced. By bridging tumor antigens with T-cell receptors (TCR), these Abs can direct effector T cells to leukemic targets. *Ex vivo* expansion of leukemia-specific T cells that are genetically modified to enforce the expression of a pre-selected TCR can also provide large numbers of T cells with exquisite specificity. Furthermore, early-phase clinical trials demonstrate that tumor-associated antigens can be therapeutically engaged through the enforced expression of a chimeric antigen receptor (CAR) on clinical-grade T cells.

Natural killer (NK) cells can lyse leukemia cells without prior antigen-specific priming. Methods to enhance NK-cell cytotoxicity by use of cytokines, such as IL-2 and IL-15 or anti-killer inhibitory receptor (KIR) Abs to block inhibitory KIRs on NK cells are available to potentiate their anti-leukemia activity. Other strategies include the depletion of regulatory T cells that have increase immunesuppressive effects in acute leukemia. Furthermore, the use of bispecific Abs composed of two fragments of mAbs that bind, e.g., CD16 on NK cells and blast-specific antigens, can greatly potentiate antibody-dependent cell-mediated cytotoxicity, a major mechanism for elimination of leukemic cells.

One mechanism of immune suppression operating in cancer involves immune cell intrinsic checkpoints that are present on the surface of activated T cells. Several such checkpoint molecules serving as negative regulators of activated T cells are known, including cytotoxic T-cell antigen-4 (CTLA-4) and programmed death-1 (PD-1). Chronic overexpression of checkpoint molecules in leukemia results in T-cell dysfunction and impairs anti-leukemia tumor immunity. Clinical trials with PD-1 inhibitors to enhance T effector cells in acute leukemia patients are currently in progress.

Over the last five decades allo-HCT has been consider the model of effective immunotherapy for acute leukemia. As new immune therapies emerge and are translated to the clinic, our perception of allo-HCT is likely to change. The availability of immune therapies alone or in combination with chemotherapy might limit indications for allo-HCT and may also complement and improve results of allo-HCT in the foreseeable future.
